# Effectiveness of YouRAction, an Intervention to Promote Adolescent Physical Activity Using Personal and Environmental Feedback: A Cluster RCT

**DOI:** 10.1371/journal.pone.0032682

**Published:** 2012-03-05

**Authors:** Richard Geuchien Prins, Johannes Brug, Pepijn van Empelen, Anke Oenema

**Affiliations:** 1 Department of Public Health, Erasmus University Medical Center, Rotterdam, The Netherlands; 2 EMGO Institute for Health and Care Research and the Department of Epidemiology & Biostatistics, VU University Medical Center, Amsterdam, The Netherlands; 3 Department of Prevention and Health Care, TNO (Netherlands Organisation for Applied Scientific Research) Quality of Life, Leiden, The Netherlands; 4 Department of Health Promotion, Maastricht University, Maastricht, The Netherlands; University College London, United Kingdom

## Abstract

**Background:**

In this study the one and six months effects of the computer-tailored YouRAction (targeting individual level determinants) and YouRAction+e (targeting in addition perceived environmental determinants) on compliance with the moderate-to-vigorous physical activity (MVPA) guideline and weight status are examined. In addition the use and appreciation of both interventions are studied.

**Methods:**

A three-armed cluster randomized trial was conducted in 2009–2010 with measurements at baseline, one and six months post intervention. School classes were assigned to one of the study arms (YouRaction, YouRAction+e and Generic Information (GI) control group). MVPA was derived from self-reports at baseline, one and six months post intervention. Body Mass Index and waist circumference were measured at baseline and six months post intervention in a random sub-sample of the population. Use of the interventions was measured by webserver logs and appreciation by self-reports. Multilevel regression analyses were conducted to study the effects of the intervention against the GI control group. ANOVA's and chi-square tests were used to describe differences in use and appreciation between study arms.

**Results:**

There were no statistically significant intervention effects on compliance with the MVPA guideline, overweight or WC. Access to the full intervention was significantly lower for YouRAction (24.0%) and YouRAction+e (21.7%) compared to the GI (54.4%).

**Conclusion:**

This study could not demonstrate that the YouRAction and YouRAction+e interventions were effective in promoting MVPA or improve anthropometric outcomes among adolescents, compared to generic information. Insufficient use and exposure to the intervention content may be an explanation for the lack of effects.

**Trial Registration:**

TrialRegister.nl NTR1923

## Introduction

Promoting physical activity (PA) among adolescents is a public health priority [1], [2], especially because most adolescents in the Netherlands – like elsewhere [3], [4], [5], [6] do not meet the guideline of being moderate-to-vigorously physically active (MVPA) for at least one hour each day [7]. Additionally, various studies show that PA further declines during adolescence [8], [9], [10], [11], [12], [13] and tracks to a certain extent from adolescence to adulthood [14]. In sum, there is an indisputable need to promote PA among adolescents.

PA levels can be promoted if interventions target important and modifiable determinants, use theory and evidence based behaviour change methodologies and fit with the target group. Current evidence [8], [15], [16] and theoretical insights [17] suggest that motivational (e.g. intention to be active and attitude towards PA) as well as environmental level determinants (e.g. availability of facilities to be active) should be addressed in interventions to improve PA among adolescents. It is likely that there is a large variability in PA levels, awareness of one's own PA level, attitude, self-efficacy, intention to change and opportunities to be active in the residential neighbourhood among adolescents. In promoting PA it is, therefore, important to take these individual differences into account and to adapt the educational content to the behaviour, circumstances and beliefs of each individual adolescent. Computer tailoring is a health education technique that makes such an adaptation of information to individual characteristics possible. Various reviews show that computer-tailoring is a promising technique to promote health behaviours [15], [18], [19], [20] and also PA among adolescents [21]. In addition, it has been shown that compared to information delivered through traditional or print information, computer-based information delivery suited adolescents better [22]. Therefore, a web-based computer-tailored intervention would be a promising intervention strategy to promote PA among adolescents. While socio-ecological models indicate that both individual and (physical) environmental factors are determinants of PA, to date most computer-tailored interventions solely provided tailored feedback on individual level determinants. To promote MVPA among adolescents and to gain insight in the additional effect of incorporating environmental feedback in computer-tailored interventions two versions of the computer tailored Youth of Rotterdam in Action (YouRAction) intervention were developed [23]. The basic YouRAction intervention targets individual level determinants, whereas the YouRAction+e intervention targets individual and perceived environmental determinants (e.g. awareness of availability of sports facilities in the residential neighbourhood). Both versions of the intervention are web-based and developed for use in school classes. The present paper describes the evaluation of the effects of the comprehensive YouRAction interventions as compared to generic information among Dutch adolescents.

The technique of computer tailoring facilitates adaptation of information to individual characteristics and is, therefore, expected to be effective for all adolescents regardless of their gender, ethnic background and educational level. Recently, it has been shown that a computer-tailored intervention aimed to increase PA among Flemish adolescents was indeed equally effective across educational groups [24]. Nevertheless, it remains important to evaluate whether tailored interventions are equally effective in relevant subgroups.

The aims of this study are to 1) evaluate the effectiveness of the YouRAction and YouRAction+e interventions on compliance with the MVPA guideline and minutes per day spend in MVPA among adolescents in the first year of secondary education in a cluster randomized trial with a generic information (GI) control group 2) evaluate the effectiveness of the two interventions on overweight and waist circumference (WC) in a sub-sample of the study population, 3) explore differential intervention effects for boys and girls, adolescents attending higher and lower levels of education and adolescents with a Western or non-Western ethnic background, and 4) describe use and appreciation of the interventions [25].

We hypothesized that after exposure to the intervention

1. in the YouRAction and YouRAction+e groups compliance with the MVPA guideline is 10% higher as compared to the GI control group at one and six months post-intervention and that average daily minutes spend in MVPA will be higher in the YouRAction and YouRAction+e groups;
2. the sub-sample of adolescents who are allocated to the intervention groups will be more likely to have a normal weight and WC compared to the GI group at six months post-intervention;

## Methods

### Design

A three-armed cluster randomized controlled trial was conducted in 2009–2010 with measurements at baseline, one and six-months post-intervention. School classes (clusters) were randomly assigned to one of the study arms (i.e. YouRAction, YouRAction+e, GI), in a computer determined sequence. Randomization was done in blocks of nine classes, to ensure that equal numbers of classes were assigned to each study arm. The random allocation sequence was concealed until the study arms were assigned.

Data on demographics, PA behaviour and determinants were collected at all time points by means of questionnaires administered during a school hour. Completion of the questionnaires was supervised through a research assistant (who was blinded to group assignment) and a teacher. Height, weight and WC were measured by a trained research assistant at baseline and six months post-intervention in a sub-sample of the study population.

The Medical Ethics Committee of the Erasmus Medical Center approved this trial. This trial is registered in the Netherlands Trial Registry (NTR1923). The protocol for this trial and supporting CONSORT checklist are available as supporting information; see Checklist S1 and Protocol S1.

### Recruitment of participants and procedure

Participants were adolescents aged 12–13 years, in their first year of secondary school, since this was the target group of the interventions. As a first step in recruitment, the health coordinators of 69 schools in the area of Rotterdam (the Netherlands) were contacted by phone. If they were interested in participating, a brochure with more detailed information about the intervention content and the research procedure was send to the schools and a member of the research team visited the schools for further information exchange and planning. In each participating school between 1 and 12 classes (depending on the size of the school), in which regular secondary education was given, were selected for participation. All adolescents in the selected classes were invited to take part in the study.

Based on a sample size calculation, 17 classes with on average 22 adolescents per class would be needed in each study arm, to detect a 10% difference in compliance with the MVPA guideline at six months post-intervention, compared to the GI group (alpha = 0.05, power = 0.80, ICC = 0.02, expected %compliance with the MVPA guideline in the GI group = 15%).

In total 54 classes, from 12 schools, in Rotterdam and surroundings were recruited. Prior to the baseline measurement, adolescents and their parents received detailed information about the trial. Based on this information, the adolescent and his/her parent or carer could decide to decline participation in the trial by returning a written objection form. Of the 1240 adolescents, 27 (2.2%) declined to participate in the study; 1213 adolescents were included in the trial.

### Measures

The aim of the interventions was to promote compliance with the MVPA guideline by 10%. Following the previously published protocol [23], this is assessed by the primary outcome measures compliance with the MVPA guideline and minutes spent in MVPA, based on self-reported PA behaviour. Secondary outcome measures were objectively measured PA and objectively assessed BMI and waist-circumference.

#### Primary outcome measures: Compliance with MVPA guideline and minutes spent in MVPA

Compliance with the MVPA guideline and minutes spent in MVPA was calculated for all three time points (baseline, one month and six months post-intervention).

Compliance with the MVPA guideline was assessed by using one item of the PACE+ sixty-minute screening measure for MVPA “Over the past 7 days, on how many days were you physically active for a total of at least 60 minutes per day?”; 0–7; test-retest ICC = 0.72, r: 0.37, p<0.01 for correlation with accelerometer [26]). This measure was dichotomized into compliance (1; i.e. 7 days at least 60 minutes in MVPA) vs non-compliance (0; i.e. less than 7 days at least 60 minutes in MVPA).

Minutes spent in MVPA was assessed with an adapted version of the Activity QUestionnaire for Adolescents & Adults (AQUAA; test-retest ICC = 0.54 for moderate-to-vigorous activities, r = −0.23, p>0.05 for correlation with accelerometer) [27]. In the adapted version of the questionnaire the frequency (in days per week) and duration (in minutes per day) of walking and cycling to school, walking and cycling in leisure time and participation in sports during the past 7 days were assessed. A MET-score was attached to all activities, based on the compendium of Energy Expenditures for Youth [28], assuming that activities were done at moderate effort. PA of more than 10 hours per day was considered to be unrealistic and therefore these values were re-coded to missing values. A MVPA score was created by summing the average daily minutes of activities higher than 5 MET, as recommended by the Dutch MVPA guideline [29]. Because data were skewed (skewness ranged from 3.51 to 3.85), the log transformed (natural logarithm) variable was used in the analyses.

#### Secondary outcome measures: Anthropometrics and accelerometers

At baseline and six months post-intervention, body weight, body height and WC were measured by trained research assistants in a random subsample (40% of total sample) of adolescents. A calibrated electronic scale (SECA 888) was used to measure body weight with an accuracy of 0.2 kg. A portable height rod (SECA 225) was used to determine body height to the nearest 0.1 cm. Adolescents were measured in their underwear, without shoes. At each measurement height was measured twice and the average value was considered to be the height of the adolescent. Body Mass Index (BMI) was calculated by using the measured height and weight (kg/m^2^). BMI cut points for adolescents, as defined from the International Obesity Task Force (IOTF), were used to categorize adolescents in “normal weight” (including underweight), “overweight” and “obese” [30].

WC was measured twice to the nearest 0.1 cm, using SECA 200 circumference measuring tapes. If there was more than 1.0 cm difference between the two measurements, two additional measurements were taken. The average of the last two measures of WC was used as the measure for waist circumference.

At baseline, one month and six months post intervention a random 10% subsample of adolescents was asked to wear an accelerometer (Actigraph GT3X) on his or her right hip, for one week, to measure physical activity. Due to high loss of accelerometer data (mainly due to non-wear), too few (i.e. 15) adolescents had full data; therefore it was decided not to conduct analyses and present results for the accelerometer data.

#### Demographics

At baseline, questions on date of birth (to calculate age), gender, country of birth of the adolescent and both parents (to determine ethnic background), and the level of education that the adolescent attended (i.e. lower vocational education (lower education) or secondary education preparing for further college or university training (higher education)) were included. Ethnicity was defined according to the procedures of Statistics Netherlands; an adolescent was considered to be of Western background if both parents were born in Europe, North America, Oceania, Indonesia or Japan. If at least one parent was born elsewhere, the adolescent was considered to be of non-Western background [31]. Hence, a dichotomous measure of ethnicity was constructed: Western background (1) vs non-Western background (0).

#### Process evaluation measures on use and appreciation

To assess self-reported exposure to the intervention, adolescents in the YouRAction, YouRAction+e or GI groups reported in how many lessons they had used their assigned intervention (none (0), 1 (1), 2 (2), 3 (3), more than 3 (4)). Additionally adolescents were asked if and how often they had made the YouRAction were obtained on homework assignment (no (0), yes, once (1), yes, twice (2), yes, three times (3)). This latter variable was dichotomized into ‘none’ (0) versus “made at least one homework assignment” (1). Objective data on intervention usage were obtained from web server logs (i.e. log in frequency, modules accessed). Data were obtained for the number of times an adolescent (attempted) to log-in and for access to the first page of each lesson.

In the one month post-intervention questionnaire process measures appreciation, personal relevance, quality, usefulness and usability of the intervention content, as well as technical problems encountered when accessing the intervention (Appendix S1).

### Interventions

The YouRAction interventions are web-based computer tailored PA promotion interventions which were systematically developed based on the Intervention Mapping protocol [32]. These interventions were meant for use in a school-based setting, mainly as part of class room activities. Teachers were instructed by research staff on how to implement the intervention and they received a teacher manual to assist them in implementing the intervention. The YouRAction interventions consisted of three sessions that could be worked through during three lessons, and homework assignments that were provided after the second and third lesson. All adolescents in one class logged in to the website simultaneously and worked through the class assigned intervention individually. Hereafter the YouRAction interventions will be briefly described; a more thorough description is published elsewhere [23].

#### YouRAction

All three lessons consisted of one or more self-regulatory phases (i.e. monitoring, motivational, goal setting, active goal pursuit and evaluation phases). In the first lesson the focus was on improving knowledge about MVPA and how much activity adolescents should engage in. Subsequently awareness of one's own PA level was increased (monitoring phase). In the second and third lesson the adolescents were motivated (by targeting attitudes, self-efficacy, subjective norm) to make a change in one of the PA sub-behaviours (active transport, leisure time activity or sports), depending on the feedback on their personal PA level (motivational phase). Subsequently adolescents could state a goal and form an action plan for how they wanted to improve their PA level (goal setting phase). In a week in between two lessons adolescents could evaluate whether they had enacted their plans and achieved their goals (phase of active goal pursuit). They could also make plans for how to deal with difficult situations they had encountered and state a new goal (evaluation phase). Most elements in the YouRAction intervention were theory based and translated in written feedback, cartoons, quizzes and web-movies.

#### YouRAction+e

The content of the YouRAction+e is identical to the basic YouRAction intervention, but in addition provides feedback on the availability of PA facilities in the residential neighbourhood of the adolescent via GoogleMaps. This was done by displaying facilities to be active with icons in GoogleMaps; adolescents could click on these icons to get more extensive information about the facility (e.g. website, specific information about sports that could be done).

The YouRAction interventions were pilot tested among 133 adolescents from 7 classes on comprehensibility and usability. During this pilot test performance problems were observed (slow website), when a whole class used the intervention. These problems were resolved by the software developers by installing a web server with higher capacity and optimization of processing of tailoring algorithms.

#### GI

The GI group received a non-tailored website containing general information on PA and healthy eating. This website was designed for 3 lessons and was also implemented in a class setting by teachers. The visual design of this website was identical to the design of the YouRAction and YouRAction+e interventions. This intervention was also called YouRAction.

### Statistical Analyses

Multinomial regression analyses were used to check equality of study groups for adolescents with baseline data. In these regressions, intervention condition was the dependent variable and gender, ethnicity, education and compliance to the MVPA guideline were the independent variables.

Logistic regression analyses were performed to study whether there was selective drop-out at first and second follow-up assessment. Drop-out (in analysis (1)/not in analysis (0)) was regressed on demographics (i.e. gender, age, ethnicity, education), intervention group and compliance with the MVPA guideline at baseline.

The effectiveness of YouRAction and YouRAction+e was studied by means of multilevel logistic (for compliance with the MVPA guideline, % overweight) and linear (for average minutes of MVPA, WC) regression analyses, taking possible clustering of students in school classes into account. Separate analyses were performed for the one and six month assessments. The outcome variables assessed at one and six months post-intervention (i.e. compliance with the MVPA guideline, minutes spend in MVPA, body mass index and waist circumference) were regressed on two dummy variables, indicating the YouRAction and YouRAction+e interventions, with the GI as the reference group and the baseline value of the outcome measure under study. The analyses were further adjusted for demographic factors that significantly differed between study arms at baseline.

Subsequently it was explored whether gender, ethnicity or level of education moderated the effects of YouRAction and YouRAction+e on compliance with the MVPA guideline and daily minutes spend in MVPA. This was done by adding, for both interventions simultaneously, an “intervention dummy * demographic factor” interaction term to the regression analysis. If these interaction terms were statistically significant at p<0.10, stratified analyses were conducted.

Complete case and intention-to-treat with last observation carried forward analyses were conducted. The data and results from the complete case analyses are presented in the tables.

ANOVA's (continuous outcomes) and chi-square tests (dichotomous outcomes) were used to describe group differences in use of the assigned intervention. For adolescents who used their assigned interventions at least once, appreciation of the intervention was also assessed with ANOVA's and chi-square tests.

Except for interaction terms, results with a p-value lower than 0.05 for a two-sided test were considered to be statistically significant. All analyses were conducted in STATA 11.0.

## Results

### Participants

**Figure 1:**
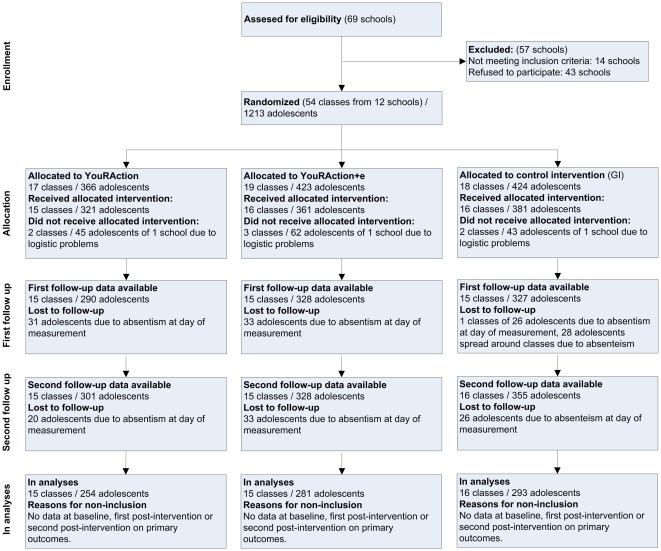
Flow diagram of the selection and enrolment of the study participants.

A total of 12 schools with a total of 54 classes and 1213 adolescents participated in the study (Figure 1). In one school seven classes dropped out after allocation, due to logistic problems at that school. No evidence for selective drop-out between baseline and first follow-up was found. However, adolescents in vocational classes and boys were more likely to have dropped out at the second follow-up. Table 1 shows background information of the participants included in the complete case analyses. The proportion of students attending lower level education was significantly lower in the GI group and all analyses were therefore adjusted for level of education.

### Intervention effects

**Table 1:** Baseline class and adolescent characteristics of adolescents in complete case analyses for the YouRAction, YouRAction+e, GI and total group.

	YouRAction	YouRAction+e	GI	Total group
**Class factors**				
Number of classes	15	15	16	46
**Individual factors**				
Number of adolescents	254	281	293	828
Age (SD)	12.7 (0.5)	12.7 (0.5)	12.6 (0.4)	12.7 (0.5)
School level (%lower level education)	56.3%	56.9%	19.8%	43.6%*
Ethnicity (% non-Western)	25.2%	22.1%	17.7%	21.4%
Gender (% male)	52.8	50.9	53.4	52.4%
Compliance with MVPA guideline (% compliant)	17.3%	15.3%	12.6%	15.0%
Minutes MVPA (SD)	126.1 (142.1)	117.3 (104.4)	134.9 (125.6)	126.1 (124.4)

SD = standard deviation; GI = general information;

* = p<0.05; derived from multinomial regression analyses.

Table 2 shows that there were no statistically significant group differences on compliance with the MVPA guideline or minutes spend in MVPA at any of the post-intervention measurements. Table 2 further shows that there were no statistically significant effects of the YouRAction and YouRAction+e interventions on prevalence of overweight including obesity, and WC at the six months post-intervention measurements. Intention-to-treat analyses resulted in similar, non-significant results.

**Table 2:** Baseline and post-intervention mean scores (SD) or percentages for compliance with the MVPA guideline, minutes spend in MVPA, weight status and waist-circumference and unstandardized regression coefficients from the regression analyses with GI group as reference.

	Percentages and Means (SD)			Unstandardized regression coefficients (95% CI)†	
	YouRAction	YouRAction+e	GI	YouRAction vs GI	YouRAction+e vs GI
*Primary outcomes (N/k)*	254/15	281/15	293/16		
Compliance with MVPA guideline					
Baseline	17.3%	15.3%	12.6%		
One month post-intervention	11.8%	15.7%	14.3%	−0.30 (−0.84;0.25)	0.11 (−0.40;0.61)
Six months post-intervention	13.0%	15.7%	18.8%	−0.42 (−0.99;0.15)	−0.16 (−0.70;0.38)
Minutes in MVPA					
Baseline	126.1 (142.1)	117.3 (104.4)	134.9 (125.6)		
One month post-intervention	95.9 (79.4)	105.5 (96.8)	109.1 (90.1)	−0.03 (−0.16;0.10)	0.08 (−0.05;0.20)
Six months post-intervention	108.1 (109.5)	115.0 (90.6)	111.5 (92.6)	0.01 (−0.14;0.17)	0.07 (−0.08;0.23)
*Secondary outcomes (N/k)*	118/15	136/16	132/17		
Overweight or obese					
Baseline	16.1%	20.6%	14.4%		
Six months post-intervention	16.1%	20.6%	13.6%	0.16 (−1.01;1.33)	0.28 (−0.86;1.42)
WC					
Baseline	67.1 (7.9)	68.6 (8.7)	66.2 (7.9)		
Six months post-intervention	68.3 (8.2)	70.4 (9.4)	67.5 (7.8)	−0.38 (−1.39;0.62)	0.16 (0.82;1.15)

MVPA = moderate-to-vigorous physical activity, WC = waist circumference, GI = general information; SD = standard deviations; N = number of adolescents; k = number of classes;

* = p<0.05;

† multilevel linear or logistic regression analyses with class and individual as levels, adjusted for level of education.

### Interaction effects

Explorative interaction analyses showed that there was no statistically significant interaction between the dummy variables for intervention groups and gender or education. However, there was a statistically significant interaction between the dummy variable for the YouRAction program and ethnic background on compliance with MVPA guideline at six months post-intervention (Beta: -1.33, 90% CI: -2.59;-0.08). Stratified analyses showed that adolescents of non-Western background in the YouRAction group had lower compliance with the MVPA guideline at the six months post-intervention measurement compared to adolescents in the GI group (Table 3).

**Table 3:** Baseline and six month post intervention compliance with the MVPA guideline and unstandardized regression coefficients of the effects of YouRAction vs GI stratified for adolescents with a Western or non-Western ethnic background.

	% Compliance with MVPA guideline at baseline	% Compliance with MVPA guideline at six months post-intervention	Unstandardized regression coefficients†(95% CI)
Western background			
GI (N = 242/k = 15)	14.1%	19.0%	
YouRAction (N = 190/k = 15)	18.4%	15.8%	−0.17 (−0.81;0.46)
Non-Western background			
GI (N = 52/k = 13)	5.8%	17.3%	
YouRAction (N = 64/k = 15)	14.1%	4.7%	−1.72 (−3.18;−0.25)*

GI = general information; CI = confidence intervals; N = number of adolescents; k = number of classes;

† multilevel linear or logistic regression analyses with class and individual as levels, adjusted for level of education;

* = p<0.05.

### Use and appreciation of the interventions

The self-reported number of lessons in which the intervention was used did not differ significantly between the groups. However, webserver logs showed that adolescents in the YouRAction and YouRAction+e group signed in significantly more often than adolescents in the GI. In total 91.3% of the adolescents in YouRAction accessed the second lesson as compared to 71.9% in the GI group (p<0.01) and 78.7% in the YouRAction+e group (p = 0.02). However, access to the third lesson was significantly lower for the YouRAction (24.0%, p<0.01) and YouRAction+e groups (21.7%, p<0.01) when compared to the GI group (54.4%) (Table 4).

**Table 4:** Self-reported and objectively measured exposure to intervention content by intervention group and differences between intervention groups.

	YouRAction		YouRAction+e		GI		
	N/k	Mean(SD)	N/k	Mean(SD))	N/k	Mean(SD)	Significant differences
*Exposure in total*							
Self-reported number of school lessons	213/15	2.46 (1.29)	252/15	2.63 (1.32)	284/16	2.50 (1.22)	
Objective log in frequency	214/15	2.49 (1.44)	247/15	2.68 (1.08)	266/16	2.20 (1.03)	YouRAction>GI (p = 0.02)† YouRAction+e>GI (p<0.01)†
Made at least 1 homework assignment (self-reported)	214/15	20.1%	252/15	21.0%	282/16	24.8%	
*Objectively assessed exposure to the three lessons*							
First lesson	224/15		244/15		263/16		
Second lesson (% of visited first lesson)		91.3%		78.7%		71.9%	YouRAction>YouRAction+e (p = 0.02)YouRAction>GI (p<0.01)‡
Third lesson (% of visited first lesson)		24.0%		21.7%		54.4%	YouRAction<GI (p<0.01)‡YouRAction+e<GI (p<0.01)‡

GI = general information; SD = standard deviation; N = number of adolescents; k = number of classes;

† based on one-way ANOVA;

‡ based on chi-square tests.

With regard to most measures of appreciation of the intervention and the content of the advice and usability of the intervention content no differences between the three study arms were found (Table 5). However, adolescents in the GI group liked the intervention more than adolescents in the YouRAction group (Table 5). The YouRAction+e intervention was perceived as more personally relevant than the GI. Technical problems were more often reported by adolescents in the YouRAction group (43.0%, p<0.01) and YouRAction+e group (34.0%, p<0.01) than by those in the GI group (16.4%). More specifically, a slow intervention was a technical problem that was more prevalent among adolescents who received YouRAction (39.2%, p<0.01) and YouRAction+e (25.9%, p = 0.02) as compared to the GI intervention (13.9%) (Table 5).

**Table 5:** Appreciation of YouRAction, YouRAction+e and GI reported by adolescents who at least used the intervention once and differences between intervention groups.

	YouRAction		YouRAction+e		GI		
	N/k	mean(SD)	N/k	mean(SD)	N/k	mean(SD)	Sign. differences
School mark (1–10)	192/15	5.81 (2.19)	228/15	6.04 (1.91)	249/16	6.25 (2.01)	
Liked the program†	194/15	2.52 (1.16)	224/15	2.57 (1.11)	249/16	2.80 (1.23)	YouRAction<GI (p = 0.04)‡
Program was interesting†	193/15	2.61 (1.15)	224/15	2.62 (1.21)	248/16	2.82 (1.21)	
*Personal relevance*							
Personal relevance†	194/15	2.65 (1.05)	228/15	2.77 (1.06)	250/16	2.47 (1.06)	YouRAction+e>GI (p = 0.01)‡
Advice suited well†	194/15	2.88 (1.11)	228/15	2.90 (1.08)	249/16	2.78 (1.05)	
*Content of advice*							
Learned a lot†	192/15	2.88 (1.14)	227/15	2.88 (1.10)	249/16	2.94 (1.21)	
Advice was useful†	194/15	3.08 (1.08)	228/15	3.07 (1.07)	249/16	2.99 (1.13)	
Could adhere to advice†	194/15	3.12 (1.08)	228/15	3.16 (1.16)	248/16	3.20 (1.12)	
*Usability*							
Program was easy to use†	194/15	3.29 (1.14)	228/15	3.48 (1.19)	251/16	3.43 (1.26)	
Program was easy to understand†	194/15	3.74 (0.97)	227/15	3.72 (1.10)	249/16	3.86 (1.05)	
Faced technical problems during intervention usage (% yes)	193/15	43.0%	227/15	34.0%	250/16	16.4%	YouRAction>GI (p<0.01)§YouRAction+e>GI (p<0.01)§
*Type of technical problems*							
Could not log in (%)	194/15	8.8%	228/15	12.3%	249/16	5.2%	
Program was slow (%)	194/15	39.2%	228/15	25.9%	251/16	13.9%	YouRAction>GI (p<0.01)§YouRAction>YouRAction+e (p = 0.02)§YouRAction+e>GI (p = 0.02)§
Got stuck in program (%)	193/15	14.0%	227/15	9.7%	250/16	4.8%	

GI = general information; SD = standard deviation; N = number of adolescents; k = number of classes;

† 5 point scale (1–5);

‡ based on one-way ANOVA;

§ based on chi-square tests.

## Discussion

In contrast to our hypothesis and results reported earlier in the scientific literature [15], [16], [21] we could not demonstrate that the computer-tailored YouRAction and YouRAction+e interventions were more effective than a general information intervention in promoting MVPA or weight related outcomes among adolescents; in explorative analyses some evidence was even found that YouRAction had a negative effect on compliance with the MVPA guideline for adolescents with a non-Western ethnic background. However, the results on negative effects among non-Western adolescents should be interpreted with care, as the low absolute numbers of non-Western adolescents complying with the MVPA guideline may have caused instable results. If confirmed in other studies, future interventions should address these potential differences in intervention effects with regard to ethnicity. Our results showed that the exposure to the tailored interventions was lower than intended and significantly lower than the GI group, which may have affected the potential for finding an effective intervention [25]. Because of the self-regulatory structure of the interventions, the hypothesized effect was dependent on repeated exposure to the intervention. Therefore, it is unclear what the potential effectiveness of a theory and evidence based computer-tailored intervention is under more favourable implementation conditions and what the added effect of environmental feedback can be.

The tailored interventions were developed according to the Intervention Mapping protocol [32], which facilitates that the intervention is strongly rooted in behaviour change theory. The interventions included a large number of theory based change strategies and were rather extensive. Participants had to complete a substantial number of questions to receive sufficiently tailored feedback on a range of potential behavioural determinants. As a result considerable amounts of data needed to be loaded when adolescents logged in to the website. When many adolescents worked simultaneously on the YouRAction interventions, this caused severe load on the server, resulting in a slow intervention program and log-in problems. Perceived slowness was significantly more prevalent in both YouRAction interventions. The YouRAction interventions were tested extensively before broader implementation, but the problems with regard to higher number of participants evidently only appeared when the intervention was used on a larger scale. It is, therefore, recommended that stress-testing of a program to assess performance of a web-based intervention when multiple people log-in simultaneously are performed in addition to pre-tests in the setting in which the intervention takes place.

Besides sub-optimal implementation, factors in the intervention and study design may have further limited the detection of potential intervention effects. First, the evidence for most of the methods used to modify determinants is derived from studies among adults and there is only limited evidence as to how effective these specific strategies are for an adolescent target group. Most of these methods were based on theory [32], [33], but no empirical evidence is available for their effectiveness when incorporated in a computer-tailored intervention developed for adolescents. Future studies need to examine which methodologies are effective in computer-tailored intervention for adolescents. Secondly, in the design of our evaluation study, the use of a control group that received an intervention is likely to have impacted our findings. It has been shown that effect sizes tend to be lower for computer-tailored interventions that were tested against a generic information control group as compared to a no-intervention control group [20]. However, the design we used allows drawing conclusions about the effects of tailoring and not only about the effects of an intervention as such.

Despite the fact that we could not demonstrate the effectiveness of the YouRAction and YouRAction+e interventions, we think it is too early to suggest that the content of the programs or tailoring is ineffective; rather efforts are needed to optimize the implementation and use of these interventions. Therefore, we recommend pursuing research on tailoring and more specifically tailoring with environmental feedback to promote PA among adolescents. The effectiveness of such interventions has not been evaluated a lot, and their effectiveness is relatively unknown but the technique has always been indicated as promising also in other studies [21]. The incorporation of feedback on opportunities in the neighbourhood environment to be active was a unique feature of YouRAction+e. This strategy was found to be promising in supporting the promotion of PA among an older adult sample [34], [35], [36]. However, this strategy has not been applied before in a web-based program for adolescents. Interestingly, the YouRAction+e intervention was perceived as more personally relevant and showed higher point estimates than the YouRAction intervention. Future studies aimed at promoting PA among adolescents should thus consider the incorporation of environmental feedback in tailoring.

Strengths of the present study include the cluster randomized design and the use of measured anthropometrics in a sub-sample of the study population. Also the use of objective measures on exposure to the interventions is a strength of this study, where previous studies called upon [16]. The use of a self-reported measure of PA is a limitation, as this may be prone to information and social desirability bias. It may be that adolescents who received one of the YouRAction interventions were more likely to report their PA more realistically (and thus lower [37]) at the follow-up measures than the GI students, because they received tailored feedback about their levels of PA. Using objective measures of PA, such as accelerometers, may give insight into this; we planned to use accelerometers, however due to the high levels of data loss it was not possible to draw conclusions on the accelerometer data.

In conclusion, we could not demonstrate an effect of the theory and evidence based YouRAction and YouRAction+e interventions in promoting PA or more favourable anthropometric outcomes among adolescents. One of the explanations for a lack of effect may be insufficient use and exposure to the intervention content, due to technical problems. Since the use of environmental feedback in computer tailored PA interventions seems promising, further research is needed to evaluate the potential effect of such a component. Furthermore, future studies should use more objective measures of PA to evaluate the effectiveness of the interventions.

## Supporting Information

Protocol S1 Trial Protocol.
(DOC)

Checklist S1 CONSORT Checklist.
(DOCX)

Appendix S1 Description of process evaluation measures.
(DOC)
