# Supplementary material for: Effectiveness of YouRAction, an Intervention to Promote Adolescent Physical Activity Using Personal and Environmental Feedback: A Cluster RCT
Source: PLoS One. 2012 Mar 5;7(3):e32682. doi: 10.1371/journal.pone.0032682 (PMC3293840; doi:10.1371/journal.pone.0032682)
Supplement: Protocol S1 — Trial Protocol. (DOC) [file pone.0032682.s001.doc]

**Protocol S1**

**Physical activity promotion in adolescents: Development and evaluation of interventions tailored to personal motivations and environmental opportunities**

## Funded by ZonMW

Prins, R.G.

Oenema, A.

Beenackers, M.A.

Mackenbach, J.P.

Brug, J.

**Introduction**

Sufficient physical activity (PA) is associated with health benefits for adolescents, like a lower risk for becoming overweight or obese ^1^, higher bone density ^1, 2^, less depressive symptoms ^1^ and healthier cardiovascular risk profiles ^2^. These health benefits may also track into later life. Furthermore, physically active adolescents are more likely to become physically active adults ^3^. However, a majority of Dutch adolescents do not meet the recommended minimum levels of PA. Only one out of four Dutch adolescents meet the PA guideline of engaging in moderate intensity physical activity for at least one hour each day ^4^ and one out of three adolescents engage in sufficient higher intensity sports activities ^5^. Similar figures have been found in other Western countries ^6-10^.

There is an urgent need for effective interventions to increase PA levels among adolescents. Currently the number of well-designed and well-evaluated interventions aiming to increase PA levels is very limited. A pre-requisite for a successful intervention is the use of a planning model that ensures that a goal directed, theory and evidence based intervention is developed. An important phase in the planned development of interventions is to identify that the most important determinants of PA and to select effective methods and techniques to modify these determinants. Besides cognitive and motivational determinants (e.g., attitude, perceived behavioral control, intention, planning), recently the physical (i.e. built) environment has been suggested to be a potent determining factor for PA among adolescents and adults ^11^. Therefore, interventions should target motivational as well as environmental determinants of PA.

Computer tailoring is one of the most promising health-education techniques for improving a variety of health related behaviors. The computer tailoring technique enables the provision of individualized, personally relevant feedback and advice ^12, 13^. Reviews of the literature and recent original investigations show that computer-tailored health education is appreciated better and may result in better awareness, stronger motivation to change as well as healthier behavior than generic health education ^12, 14-16^. Computer-tailored health education may be especially suited to address complex health-related behaviors such as physical activity.

The present study aims to evaluate the effects of two carefully planned, theory and evidence based PA interventions (YouR Action) that use computer tailoring as the main health education technique. One version of the intervention aims at modifying the motivational determinants and improving the use of self-regulation techniques (from now on called ‘motivational’ intervention), the other also incorporates environmental information in the tailored feedback (from now called ‘environmental intervention’). A specific aim of this study is to investigate the additional effects of incorporating strategies to change perceptions of the physical environment on PA behavior in the intervention. The interventions are delivered over the Internet and are meant for use in schools for secondary education.

**Description of the intervention**

The motivational and environmental interventions are developed from the same theory and evidence based background. Therefore the description of the intervention applies to both interventions, unless explicitly stated.

#### Targeting different PA sub-behaviors

## As has been pointed out in the introduction, there is a need to increase PA levels among adolescents. PA is a complex behavior, consisting of various sub-behaviors that contribute to the overall level of PA. Important PA sub-behaviors for adolescents are:

## active transport

- leisure time activities
- engagement in sports

These sub-behaviors will be targeted in the intervention.

## Determinants, methods and technique

The important motivational and self-regulation determinants that have been identified and have to be targeted in the intervention are knowledge, perception of risk, awareness of one’s own risk behavior, attitude, social influences and perceived behavioral control for becoming more physically active, goal setting, planning and relapse prevention.

The important and changeable environmental factors that have been identified are perceptions of the availability of parks, sports facilities, bicycle lanes and sidewalks.

A variety of methods and strategies such as persuasive communication, normative feedback, improving skills by showing sub-skills by an animation figure will be used to modify the motivational and self-regulation determinants. For modifying perception of the environment, interactive geographic maps will be incorporated in the intervention. By using these road maps, participants get to know where to be active in their neighborhood.

All these strategies will be combined in the Internet-delivered interventions, that will consists of four separate sessions of 35-45 minutes each. A flow scheme of the program is depicted below.

**Intervention elements**

Module 1a: general introduction to PA

Module 1b: Monitoring of PA behavior + feedback

Module 2a: active transport

Module 2b: leisure time PA

Module 2c: sports

Module 3: Working on achieving behavioral goal

Module 4: Evaluation of behavioral goals

Figure 1. Flow-chart of the interventions

#### Description of the intervention contents

The intervention starts with a general introduction to PA and the intervention, (figure 1, module 1a). The adolescents can pick an animation figure which will guide them through the intervention. Then the program will test and improve the knowledge of the adolescents about various types of PA and how much time an adolescent should spend in PA each day, by means of quizzes and feedback on the answers given. In the next step the intervention will aim at insight in an adolescents’ own PA behavior and identify areas for improvement, by means of the provision of personal, normative and comparative feedback (figure 1, module 1b). Adolescents have to complete a PA screening questionnaire, after which individualized feedback is provided on the time spent in the various PA sub-behaviors and how this compares to PA norms and PA levels of peers. Subsequently an adolescent can choose on which of the sub-behaviors (i.e. active transport, leisure time PA or sports) he or she wants to improve (figure 1, modules 2a-2c). The next phase of the program will assist the adolescent in setting goals for improving PA and sub-behaviors and in choosing and planning specific activities in order to achieve the goal, as well as teach them skills to be able to become more active. The adolescents will learn and exercise skills through interactive assignments, videos, simple text and pictures and will make detailed plans for the activities they will employ in the upcoming week, in order to achieve their goals. Furthermore, awareness of places where adolescents can engage in physical activity in their home environment will be improved by showing appropriate and safe locations in GoogleMaps, in the environmental intervention, and by giving them the task to look around in their own neighborhood for appropriate and safe locations in the motivational intervention. In the third phase of the intervention the adolescent will try to execute the plan. After this week, adolescents will log on to the website again and fill in how they performed. The program will give feedback on the progress and give practical tips to maintain or improve behavior (figure 1, module 4). After this evaluation, adolescents can choose to work further on the already chosen sub-behavior or try to work on another sub-behavior. Hence, each adolescent will get personalized feedback. The program will be delivered in school, once a week, in a four week period.

Each adolescent will receive a unique username and password. For security, these passwords are saved encrypted. To ensure maximum security, an SSL-connection is made to the website. SSL-connections ensure that data send from the website is encrypted, so this data can only be read by the person using the website. Other parties trying to tap data send from the website will not be able to read this data.

#### Pre-test and formative evaluation

During intervention development, the intervention is carefully pre-tested, to ensure that the developed materials are relevant and attractive to the target group.

**Effect evaluation**

**Aims of the study**

- To establish the short (1 month) and medium term (6 month) effects of a motivational and environmental computer-tailored intervention on PA and mediating variables among adolescents and to identify differences in efficacy between the two computer-tailored interventions among adolescents living in environmentally contrasting areas (i.e., ‘green’ versus ‘grey’) of Rotterdam and the Hague;
- To study differential intervention effects according to socio-economic status and ethnic background;
- To study whether intervention effects depend on objectively measured characteristics of the built environment;

**Design**

This study is designed as a school-based cluster randomized trial, with assessments at baseline, one month post intervention and six months post intervention. There are three study arms:

- a motivational computer-tailored-intervention,
- an environmental computer-tailored-intervention
- and a control group, which receives a standard website on physical activity, and will be given access to the most effective intervention after the end of the study.

School classes will be the unit of randomization.

Graphical depiction of the design.

R O_1_ X_1_ O_4_ O_7_

R O_2_ X_2_ O_5_ O_8_

R O_3_ C_1_ O_6_ O_9_

In which observations are depicted with an O and the interventions with a T. The measurements taken at the observation periods are shown in table 2.

## Main outcome measures

The primary outcome of this study is the % of adolescents with a PA level in agreement with the norms for PA. Secondary outcomes are PA in minutes, motivational and environmental determinants of PA, Body Mass Index (BMI) and waist circumference.

## Hypotheses

It was hypothesized that after the intervention

- a higher % of adolescents in both intervention arms will meet the norm of being physically active at least 60 minutes per day compared to the adolescents in the controlgroup;
- a higher % of adolescents of the environmental intervention will meet the norm of being physically active at least 60 minutes per day compared to the adolescents who used the motivational intervention;
- adolescents of the intervention groups will have a lower BMI and waist circumference in the medium-term measurement;
- the interventions will be equally effective among adolescents from different socio-economic and ethnic backgrounds;
- the environmental intervention will have stronger positive effects on PA behavior among adolescents living in the ‘grey’ neighborhoods (little green, high buildings)as compared to those living in the ‘green’ neighborhoods (a lot of green and low buildings)

## Participants

Participants for this study are adolescents who attend the first year of schools for secondary education in Rotterdam and the Hague. Sixteen-hundred and five adolescents will have to be recruited into the study. This is based on power calculations. Power calculations (power: 0.80, alpha: 0.05) indicated that 1605 adolescents (535 in each arm) are needed in order to find an increase of 10% in compliance to the norm of engaging in moderate intensity physically activity for at least one hour per day. The power calculations accounted for the multi-level design of this study. For this power calculation, it was assumed that 50% of adolescents comply to the norm at baseline. The adolescents will be recruited from 3-6 classes from 20 schools, with an average class-size of 20 students. This will result in a higher number of students recruited, accounting for loss to follow-up and non-participation.

## Recruitment

Recruitment of participant will be done by a stepwise procedure. First, schools will be invited to take part in the study. In cooperation with the schools, classes will be selected and randomly assigned to one of the study arms. From these classes adolescents will be recruited.

*Recruiting schools*

The intervention only contains environmental information of the cities of Rotterdam and the Hague. Therefore, schools have to be located within Rotterdam or the Hague. Schools will be stratified according to the built environment in which they are located (much green and low buildings versus little green and low buildings). Furthermore, the intervention is mainly text-based. The texts are written for adolescents with “VMBO-t” as a minimal educational level. Therefore, only schools with VMBO-t or higher levels will be invited for participation in the study.

An invitation letter to participate in the research (appendix E1a) will be send to “zorgcoordinatoren” of schools that are eligible for participation in the study. Participant information (appendix E4a) with details about the research will be attached to this invitation letter. Based on this information, schools can make an informed decision regarding their participation in the study. Two weeks after sending the invitation letter and the information package, the researcher will contact the schools to clarify any questions and to ask them if they are willing to participate.

*Recruiting adolescents*

In the participating schools 3-6 classes (depending on the size of the school) will be randomly selected for participation in the study and these classes will be randomly assigned to one of the three study conditions. All the adolescents within the selected classes will be invited for participation in the study. Adolescents and their parents will be informed about the study by means of the participant information (appendices: E1b, E2a, E4b, E1c, E2b, E4c) at the beginning of the school year. This information will be handed to them by their tutor. Adolescents and their parents will receive the letter, information and objection forms two weeks prior to the first measurements.

*Consent*

A passive consent procedure is proposed for this study, to be able to conduct a high quality study with a sufficient number of participating schools and adolescents. Adolescents and their parents will be fully informed about the study by means of the participant information and can object against participation in the study. Thus, all the adolescents in the selected classes will participate in the study unless they or their parents have objected. This class-based approach fits best with the teaching system in the Dutch schools, since the teaching and other activities in the schools are collective class activities, as opposed to individual activities. It will further reduce the burden for the schools in obtaining the informed consent forms. The nature of this study is such that it does not place a high burden on the participants, does not enforce rules or regulation on the participants and does not use intrusive types of measurement, which we believe would make a passive consent procedure appropriate. Using other than active informed consent procedures for school-based studies is important, since previous experiences, also those documented in the literature, have shown that when an active informed consent procedure is chosen participation rates are very low, which is detrimental to the study quality and generalizability of the results ^17, 18^. Non-participation is in most cases not due to active consents of non-participation, but due to non-returned consent forms, or negative group processes that occur in classes. The difficulty with receiving the completed informed consent forms also places an extra burden on the schools, which may make them decide not to participate in future studies.

## Measures

# Instruments

Electronic self report questionnaires will be used to get a good quality assessment of physical activity and physical activity sub-behaviors, cognitive and environmental determinants of physical activity sub-behaviors and demographics. The questionnaire is based on existing validated questionnaires for adolescents, or questionnaires that have previously been used for adolescents (appendix F1).

To obtain objective information about PA levels, accelerometer data will be collected in a random sub-sample of the total study population.

To obtain information about the secondary outcome measures BMI and waist circumference, measures of height, weight and waist circumference will be assessed in a random sub-sample of the population (table 1, figure 2).

# Contents of questionnaire

- Physical Activity (FPACQ):
  - Transport to and from school;
  - Leisure time (LT) active transport;
  - LT activities: moderate, light, sedentary;
  - Sports
- Cognitive determinants: perception of physical activity, risk perception, attitude, subjective norm, perceived behavioral control, intention, stages of change, goal setting, planning, perceived barriers.
- Perceived social environment: perceived behavior of parents and peers
- Perceived physical environment
  - Availability of specific amenities
  - Perceived safety
- Demographics:
  - Gender
  - Age
  - Ethnicity
  - 6 digit ZIP-code

See appendix F1 for a draft of the complete questionnaire.

Table 1. Measurements per sample

| Sample | **Total sample** | **Anthropometrics** | **Accelerometers** |
| --- | --- | --- | --- |
| N | 1605 | 300 | 400 |
| Measures | Questionnaires | Questionnaires  Anthropometrics: body weight, length, waist circumference | Questionnaires  Anthropometrics: body weight, length, waist circumference  Accelerometers |

Total sample

Acc.

Figure 2. Schematic presentation of samples

*Accelerometer data.*

To obtain an objective measure of PA behavior, accelerometers will be used in a random sub-sample of 400 adolescents (table 1, figure 2). Accelerometers are advanced pedometers, or step counters. These devices can be clipped on a belt or jeans and it records accelerations. Adolescents will be asked to wear the accelerometer for one week, except during swimming and sleeping. They will receive a careful instruction to become familiar with wearing the accelerometers.

After one week, adolescents have to hand the accelerometers to their tutor. Research staff will collect the accelerometers at school.

*Anthropometric data.*

To evaluate the effects of the intervention on determinants of health, the degree of overweight and waist circumferences are measured in a random sub-sample of 300 adolescents (table 1, figure 1). With regard to anthropometric data, a SECA scale (type: 888) to measure weight and SECA mobile measuring rod measuring-tape (type: 225) to measure height will be used. Body Mass Index (BMI) will be calculated by the following formula:

BMI = Weight/Height^2^

Besides height and weight, waist circumference will be measured using a circumference measuring-tape of SECA (type: 200).

**Procedure**

# Baseline measurements

At baseline, all students without a letter of objection of the selected classes will complete an online questionnaire during one school hour. A researcher and a teacher will be present during questionnaire completion. Furthermore, anthropometric data will be collected and the accelerometer will be distributed.

The anthropometric measures will be taken by trained research staff. Adolescents will be asked during a lesson to come in succession to a private room where the measures will be taken. To ensure privacy, anthropometric measurements will be done in a separate room with only members of the research team present. In the case that windows are present in this room, the windows will be blinded. The adolescents will be measured wearing underwear.

Adolescents with a declaration of objection are asked to do something else during the questionnaire completion.

*Intervention*

After the baseline measurements, the Your Action interventions will be implemented in the intervention classes and the generic information in the classes assigned to the control condition. The interventions and the generic information will be implemented in four regular lessons spread over four weeks. The choice of the lessons in which the interventions will be used will be decided in collaboration with the participating schools. A schoolteacher will be asked to supervise during the intervention and a research team member will visit the lessons incidentally. To ensure that the intervention is implemented properly, all teachers will receive a comprehensive manual which describes how to work with the intervention. Adolescents who declined to participate in the study will be asked to do something else during these lessons.

*Measurements one and six months post intervention*

The procedure for the measurements for the one and six months post intervention assessments will be the same as the baseline measurement. In the one month post-intervention assessment a brief process evaluation questionnaire will be added and anthropometric measurements will be left out (table 2). The process evaluation contains questions on the amount of lessons the adolescent had on PA and how they liked the intervention. Trained research staff will collect the data.

*Statistical considerations*

Multi-level logistic and linear regression analyses will be conducted using the statistical package MLWIN. Multi-level analyses allow to account for clustering of participants in different levels, for instance in schools (level 1), classes (level 2) and participant (level 3).

Differences in terms of physical activity and determinants between the different study arms will be assessed. Furthermore, interaction between the effects of the intervention and the built environment will be assessed.

# Publication of study results

Study results will be published in national and international scientific journals. Furthermore results will be communicated in national and international conferences and symposia.

## Privacy issues

All data collected in this study will be dealt with confidentially. Individual information collected in this study will be replaced by a unique code. Only the research team will get insight in the data. The METC and “de Inspectie van de Gezondheidszorg” will be provided access to the data when requested. All individual information will be dealt with according to the “WBP” and the privacy regulation of the Erasmus MC. Data will only be used for scientific research. The answers of the questionnaire, anthropometric data and accelerometer data will be processed in statistical tables in which no individuals and schools are recognizable.

## Voluntary participation

Participation in this study is voluntary. At any time, schools and adolescents can withdraw their cooperation without giving a reason. School principals, adolescents and their parents will be made aware of this in the participant information.

## Time schedule of the study

Table 2. Time schedule

|  | **Classes with extended intervention** | **Classes with basic intervention** | **Controlgroup** |
| --- | --- | --- | --- |
| May – June 2008 | Recruiting schools | | |
| June 2008 | Planning logistics together with schools | | |
| September 2008 | Informing adolescents and parents about the intervention and the research | | |
| September – October 2008 | *Baseline (O_1_)*   - Questionnaire - antropometrics subsample: weight, length and waist circumference - accelerometry subsample: accelerometric measurements during one week | *Baseline (O_2_)*   - Questionnaire - antropometrics subsample: weight, length and waist circumference - accelerometry subsample: accelerometric measurements during one week | *Baseline (O_3_)*   - Questionnaire - antropometrics subsample: weight, length and waist circumference - accelerometry subsample: accelerometric measurements during one week |
| October - November 2008 | *Intervention (X_1_)*   - Completing intervention in four weeks | *Intervention (X_2_)*   - Completing intervention in four weeks | *Intervention (C_1_)*   - Regular webbased information on physical activity |
| November – December 2008 | *One month post intervention (O_4_)*   - Questionnaire - Process evaluation - accelerometry subsample: accelerometric measurements during one week | *One month post intervention (O_5_)*   - Questionnaire - Process evaluation - accelerometry subsample: accelerometric measurements during one week | *One month post intervention (O_6_)*   - Questionnaire - Process evaluation - accelerometry subsample: accelerometric measurements during one week |
| January-April 2009 | Analyses baseline data | | |
| May – June 2009 | *Six months post intervention (O_7_)*   - Questionnaire - antropometrics subsample: weight, length and waist circumference - accelerometry subsample: accelerometric measurements during one week | *Six months post intervention (O_8_)*   - Questionnaire - antropometrics subsample: weight, length and waist circumference - accelerometry subsample: accelerometric measurements during one week | *Six months post intervention (O_9_)*   - Questionnaire - antropometrics subsample: weight, length and waist circumference - accelerometry subsample: accelerometric measurements during one week |
| July 2009 | *After last measurements*   - Intervention available for all participating schools | *After last measurements*   - Intervention available for all participating schools | *After last measurements*   - Intervention available for all participating schools |
| August 2009-September 2010 | Analyses data and reporting in national and international papers | | |

**References**
