# Supplementary material for: Effectiveness of YouRAction, an Intervention to Promote Adolescent Physical Activity Using Personal and Environmental Feedback: A Cluster RCT
Source: PLoS One. 2012 Mar 5;7(3):e32682. doi: 10.1371/journal.pone.0032682 (PMC3293840; doi:10.1371/journal.pone.0032682)
Supplement: Appendix S1 — Description of process evaluation measures. (DOC) [file pone.0032682.s003.doc]

**Appendix S1**. Description of process evaluation measures

| **Category** | **Item** | **Scale** |
| --- | --- | --- |
| Appreciation | YouRAction was fun to use | totally disagree (1) – totally agree(5) |
|  | YouRAction was interesting | totally disagree (1) – totally agree(5) |
|  | How would you rate YouRAction on a scale from 1 (worst) to 10 (best)? | 1-10 |
| Personal relevance | Advices and questions in YouRAction were specially meant for me | totally disagree (1) – totally agree(5) |
|  | The advices that the YA program gave me suited me well | totally disagree (1) – totally agree(5) |
| Content of advice | I learned a lot from YouRAction | totally disagree (1) – totally agree(5) |
|  | The advice provided by YouRAction was useful | totally disagree (1) – totally agree(5) |
|  | I could adhere to the advice | totally disagree (1) – totally agree(5) |
| Usability | YouRAction was easy to use | totally disagree (1) – totally agree(5) |
|  | YouRAction was easy to understand | totally disagree (1) – totally agree(5) |
| Technical problems | Did you experience technical problems during working with YouRAction. If so, which problems? | “No, I had no problems”, “The program was difficult to use”, “I could not log in”, “The program was too slow”, “I got stuck in the program” |
